# Validation of the 2025 ATA risk stratification system in a cohort of patients with papillary thyroid carcinoma

**DOI:** 10.1210/clinem/dgag167

**Published:** 2026-04-17

**Authors:** Claudia Moneta, Matteo Trevisan, Carla Colombo, Alessia De Luca, Marina Lugaresi, Giacomo Maria Reali, Giacomo Gazzano, Luca Persani, Laura Fugazzola, Simone De Leo

**Affiliations:** Department of Health Sciences, University of Milan, Milan 20142, Italy; Department of Health Sciences, University of Milan, Milan 20142, Italy; Department of Endocrinology, ASST Santi Paolo e Carlo, Milan 20142, Italy; Department of Endocrine and Metabolic Diseases, IRCCS Istituto Auxologico Italiano, Milan 20145, Italy; Department of Pathophysiology and Transplantation, University of Milan, Milan 20122, Italy; Department of Medical Biotechnologies and Translational Medicine, University of Milan, Milan 20133, Italy; Department of Health Sciences, University of Milan, Milan 20142, Italy; Department of Health Sciences, University of Milan, Milan 20142, Italy; Pathology Unit, IRCCS Istituto Auxologico Italiano, Milan 20145, Italy; Department of Endocrine and Metabolic Diseases, IRCCS Istituto Auxologico Italiano, Milan 20145, Italy; Department of Medical Biotechnologies and Translational Medicine, University of Milan, Milan 20133, Italy; Department of Health Sciences, University of Milan, Milan 20142, Italy; Department of Endocrinology, ASST Santi Paolo e Carlo, Milan 20142, Italy; Department of Endocrinology, ASST Santi Paolo e Carlo, Milan 20142, Italy

**Keywords:** ATA risk stratification, papillary thyroid cancer, structural recurrence or persistence, American Thyroid Association, risk factors, disease outcome

## Abstract

**Context:**

The updated risk stratification system for papillary thyroid cancer (PTC) introduced by the 2025 American Thyroid Association (ATA) guidelines has not yet been validated in a real-world setting.

**Design:**

Retrospective observational study.

**Setting:**

Tertiary care center.

**Patients:**

Data from 670 PTC patients with complete histopathological and final disease outcome were included.

**Main Outcome measures:**

We evaluated how patients previously classified by the 2015 ATA risk stratification system are redistributed according to the updated version and assessed the impact of reclassification on final disease outcome prediction.

**Results:**

The reclassification according to the 2025 ATA risk stratification showed that the proportion of “low-risk” PTC decreased by 22.2%, while “intermediate-risk” and “high-risk” PTC increased by 41.7 and 14.9%, respectively. Cross-comparison between the 2 stratification systems revealed that structural disease persistence in the 2025 “low-risk” and “high-risk” classes was comparable. The 2025 “intermediate-high-risk” class was similar to the former “intermediate-risk” class, whereas the “low-intermediate-risk” class emerged as a new class, with a risk of structural disease persistence higher than that of the 2015 “low-risk” class (*P* = .003), but lower than that of the 2015 “intermediate-risk” class (*P* = .012).

**Conclusion:**

In a real-life context, the 2025 ATA risk stratification system led to a shift toward higher-risk classes. The newly defined “low-intermediate-risk” class was the only class that significantly diverged from the classes of the 2015 stratification, with a risk of structural recurrence between the prior “low-risk” and “intermediate-risk” classes, highlighting the need for dedicated prospective studies to address proper management of this newly defined group of patients.

The ATA risk stratification system is a widely used tool for predicting disease persistence or recurrence and guiding follow-up and adjuvant treatment decisions in patients with differentiated thyroid cancer (1). Since the 2015 American Thyroid Association (ATA) guidelines (2), several studies have refined the role of some histological variables, such as tumor multifocality, particularly in tumors >1 cm, and the presence of tumor extranodal extension, as risk factors associated with increased locoregional recurrence and distant metastases (3-9). These variables, together with others such as the presence of clinically detectable lateral cervical lymph node metastasis, the presence of microscopic incomplete resection at the posterior resection margin and tumor size were included into the updated 2025 ATA risk stratification system (1). In contrast, other variables, such as cervical metastases showing radioiodine uptake on the first post-therapy whole body scan and tumor molecular profile, are no longer included in the updated stratification system (1). In particular, the genetic alterations are classified as “early genomic changes” and “events in combination with early changes associated with progression”, while recognizing that not all thyroid cancers are recommended for molecular testing.

Along with the inclusion of the new risk factors in the updated stratification system, the 2025 guidelines have also defined three separate stratifications based on tumor histotype (1 for papillary thyroid cancer (PTC) and its subtypes, 1 for follicular thyroid cancer (FTC) and invasive encapsulated follicular variant of papillary thyroid cancer (IEFVPTC), and 1 for oncocytic thyroid cancer) and expanded the number of risk classes from 3 to 4 classes (1).

In this study, we aimed to evaluate, in a real-life large cohort of PTC cases, how patients previously classified by the 2015 ATA risk stratification system are redistributed according to the updated stratification system. Moreover, we assessed the impact on the final disease outcome prediction of the new stratification compared with the previous version.

## Materials and methods

### Patients

Consecutive adult patients (>18 years old) with PTC followed at our tertiary care center and diagnosed between January 1969 and December 2024 (n = 1619) were retrospectively evaluated. Among those, patients with incomplete histopathological data required for assignment to a risk class according to either the 2015 or the 2025 ATA risk stratification systems, as well as patients for whom the final disease outcome could not be determined, were excluded from the analysis. Only patients with a minimum follow-up of 12 months were included. Patients with histological subtypes other than PTC were also excluded, including those with IEFVPTC, because this entity shares the same risk stratification as FTC in the 2025 ATA risk stratification system. Poorly differentiated thyroid cancer was also excluded, whereas high-grade PTCs were included and classified as high-risk tumors.

The clinical variables collected for each patient were gender, age at diagnosis, type of surgery, radioiodine treatment, final disease outcome, TNM, and AJCC stage according to the 8th edition. The following histological variables were also assessed: primary tumor size, presence of PTC aggressive subtypes, vascular invasion and extranodal extension, presence and type of extrathyroidal extension, status of surgical margins, location, number and dimensions of lymph node metastases, and tumor focality. Aggressive subtypes of PTC included hobnail, tall cell, columnar, diffuse sclerosing, and solid subtypes.

This study was conducted in accordance with the Declaration of Helsinki. Participants provided written informed consent. The study was approved by the Ethical Committee of the Istituto Auxologico Italiano (study code 2022_03_08_03).

### Follow-up

All patients included in the study underwent initial surgical treatment (thyroid lobectomy or total thyroidectomy, with or without central and/or lateral neck lymph node dissection), and, when indicated, subsequent radioiodine treatment.

During follow-up, periodic clinical evaluations were performed every 3-12 months, depending on disease status, and were associated with assessment of tumor markers (thyroglobulin and antithyroglobulin antibodies) and neck ultrasonography. In cases of suspected persistent or recurrent disease or evidence of disease progression, additional imaging studies were performed, including computed tomography (CT), 18F-fluorodeoxyglucose positron emission tomography (FDG-PET), and magnetic resonance (MRI).

Patients with persistent or recurrent disease received additional treatment (further radioiodine treatment, surgery, or systemic therapy), or underwent active surveillance, according to clinical judgment.

### ATA risk stratification and long-term disease outcomes

Based on histological data, each patient was assigned a risk class according to both the 2015 and 2025 ATA risk stratification systems, hereafter defined as “ATA-risk 2015” and “ATA-risk 2025,” respectively. For each patient, the disease outcome at the end of the follow-up was defined according to the response to therapy described in the 2025 ATA guidelines (1).

The end of the follow-up was defined as the date of the last visit for patients who did not undergo additional treatment beyond the initial therapy (surgery with or without first radioiodine treatment), and as the date of the second treatment for patients who received additional therapy for persistent disease.

### Comparison of the ATA-risk 2015 and the ATA-risk 2025 stratification systems

The ATA-risk 2015 and the ATA-risk 2025 were compared to assess the degree of concordance between them. Specifically, we analyzed the proportion of patients who maintained the same risk class and those who were reclassified into a different risk class.

Subsequently, a cross-comparison of final disease outcome between the risk classes of the 2 stratification systems was performed to evaluate the concordance among risk classes.

### Statistical analysis

Statistical analysis was performed by the Statistical Package for Social Sciences (SPSS) software, version 29.0.1.0. Categorical variables were described as absolute numbers and percentages. Continuous quantitative variables were reported as median and interquartile range (IQR), given their non-normal distribution determined by the Shapiro-Wilk test. For categorical variables, univariate analysis was performed using the χ2-test or Fisher's exact test, depending on the expected number of observations. We defined the *P*-value for statistical significance as <.05.

## Results

### Description of the cohort

**Figure 1:**
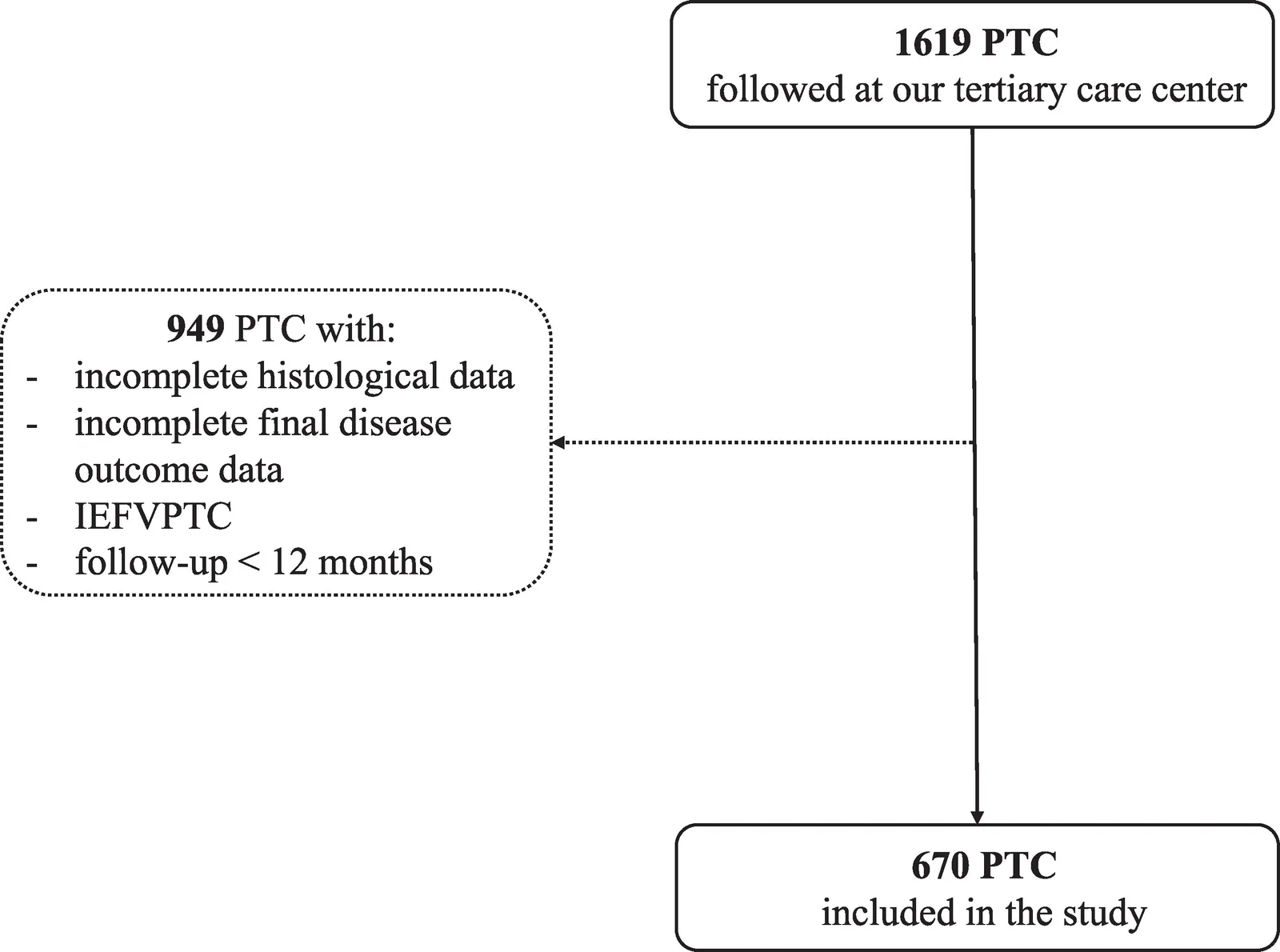
Flowchart showing the initial population and the final cohort after applying exclusion criteria.

Abbreviations: IEFVPTC, invasive encapsulated follicular variant of papillary thyroid cancer; PTC, papillary thyroid cancer.

As shown in Fig. 1, after applying the exclusion criteria, 670 out of 1619 patients with PTC followed at our center were included in the study. Patients included in the study were diagnosed between January 1990 and November 2024. In particular, 36 patients were diagnosed between 1990 and 2000, 203 patients between 2001 and 2015, and 431 patients (64.3%) between 2016 and 2024. All patients diagnosed before 1990 were excluded due to the lack of complete histopathological information required to re-assess ATA risk stratification. Patient clinicopathological features are illustrated in Table 1. In particular, 518 (77.3%) patients were female, and the median age at diagnosis was 47 years (IQR 36-57), 602/670 patients (89.9%) underwent total thyroidectomy, associated with lymph node dissection in 207 (30.9%) and radioiodine treatment in 288 (43%) patients. The use of radioiodine treatment changed over time: in the period between 1990 and 2015, 60.3% (144/239) of patients received radioiodine, whereas this rate decreased to 33.4% (144/431 patients) after 2015.

**Table 1:** Clinicopathological characteristics of the 670 patients included in the study

Characteristics	Patients (N = 670)
Gender F, N (%)	518 (77.3)
Median age at diagnosis, years (IQR)	47 (36-57)
Type of surgery, N (%)	
LT	68 (10.1)
TT	395 (59.0)
TT + L	207 (30.9)
RAIT, N (%)	288 (43.0)
Aggressive subtype, N (%)	54 (8.1)
Median tumor size at diagnosis, cm (IQR)	1.2 (0.7-1.9)
Focality, N (%)	
Unifocal	470 (70.1)
Unilateral multifocality	66 (9.9)
Bilateral multifocality < 1 cm	127 (19.0)
Bilateral multifocality > 1 cm	7 (1.0)
Extrathyroidal extension, N (%)	
Absent	494 (73.7)
Microscopic	137 (20.5)
Macroscopic	39 (5.8)
Margins, N (%)	
R0	629 (93.9)
R1	33 (4.9)
R2	8 (1.2)
R1 posterior, N (%)	10 (30.3)
Extranodal extension, N (%)	15 (2.2)
Vascular invasion, N (%)	76 (11.3)
TNM 8th edition, N (%)	
T1-T2	614 (91.6)
T3a	19 (2.8)
T3b-T4	37 (5.5)
N0/NX	549 (81.9)
N1a	65 (9.7)
N1b	56 (8.4)
M0	665 (99.3)
M1	5 (0.7)
AJCC 8th edition, N (%)	
I-II	660 (98.5)
III-IV	10 (1.5)
Median number of CC metastatic lymph nodes, N (IQR)	3 (1-6.5)
Median size of the largest CC metastatic lymph node, cm (IQR)	0.6 (0.2-1)
Median size of the largest LC metastatic lymph node, cm (IQR)	2 (0.9-3.5)
Median follow-up, months (IQR)	53.5 (21-120)

Abbreviations: CC, central compartment; F, female; LC, lateral compartment; LT, lobectomy; R0, complete tumor resection; R1, microscopic incomplete tumor resection; R2, macroscopic incomplete tumor resection; RAIT, radioiodine treatment; TT, total thyroidectomy; TT + L, total thyroidectomy and lymphadenectomy.

The tumor was multifocal in 29.9% (n = 200) of cases and bilateral in 20% (n = 134). The median primary tumor size was 1.2 cm (IQR, 0.7-1.9 cm). Microscopically and macroscopically incomplete resections occurred in 4.9% (n = 33) and 1.2% (n = 8) of cases, respectively. Among patients with microscopically incomplete resections, the posterior margin was involved in 10 of 33 cases. Extrathyroidal extension was present in 26.3% (n = 176) of cases (macroscopic in 5.8% of patients), while extranodal extension was observed in 2.2% (n = 15) of tumors. An aggressive PTC subtype was identified in 8.1% (n = 54) of tumors, and vascular invasion was present in 11.3% (n = 76) of cases. The 99.3% (n = 660) of patients were classified as stage I-II. At diagnosis, 18.1% (n = 121) of patients had lymph node metastases, whereas only 0.7% (n = 5) had distant metastases. The median number of metastatic lymph nodes removed from the central compartment was 3 (IQR 1–6.5). The median size of the largest metastatic lymph node was 0.6 cm (IQR 0.2–1.0 cm) in the central compartment and 2.0 cm (IQR 0.9–3.5 cm) in the lateral compartment, respectively.

### ATA risk stratification according to the 2015 and 2025 guidelines

**Figure 2:**
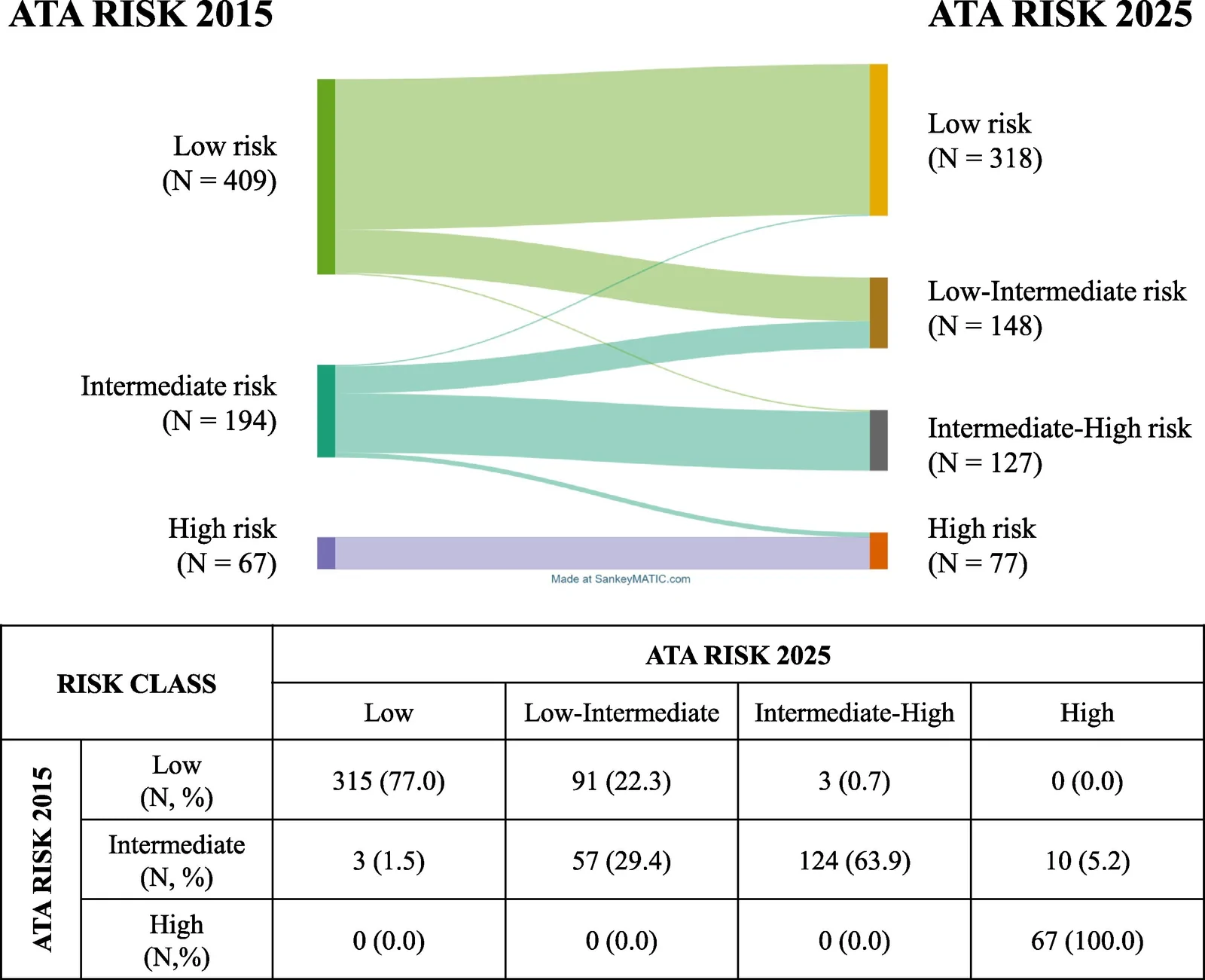
Sankey diagram showing the redistribution of patients from the 2015 ATA risk classes to the 2025 ATA-risk classes.

Abbreviation: ATA, American Thyroid Association.

As shown in Table 2, according to the ATA-risk 2015, 409 patients (61%), 194 (29%), and 67 (10%) were classified as “low-risk,” “intermediate-risk,” and “high-risk,” respectively. On the other hand, by using the ATA-risk 2025, 318 patients (47.5%) were classified as “low-risk,” 148 (22.1%) as “low-intermediate-risk,” 127 (18.9%) as “intermediate-high-risk,” and 77 (11.5%) as “high-risk” for disease persistence or recurrence. When comparing the two stratification systems, the proportion of patients classified as “low-risk” decreased by 22.2% in the ATA-risk 2025 (318 vs 409). In contrast, the proportion of patients classified as “intermediate-risk” (including the “low-intermediate-risk” and “intermediate-high-risk” classes) and “high-risk” increased by 41.7% (275 vs 194) and 14.9% (77 vs 67), respectively. The reduction in the number of patients classified as “low-risk” occurred because 22.3% (n = 91) of patients were upgraded to “low-intermediate-risk”, and 0.7% (n = 3) to “high-intermediate-risk” (Fig. 2). The reasons for the upgrade to “low-intermediate-risk” in the new stratification were: tumor size > 4 cm (n = 8), unilateral multifocal tumors (n = 33), bilateral tumors <1 cm (n = 49), and the co-presence of 2 criteria (unilateral multifocality and tumor size > 4 cm, n = 1), whereas the upgrade to “intermediate-high-risk” occurred in all patients (n = 3) due to bilateral tumor >1 cm. Only 1.5% (n = 3) of patients classified as 2015 “intermediate-risk” were downgraded to “low-risk” in the new stratification. Two patients were re-classified for lymph node metastases of 2 mm and 1 patient for lymph node uptake on the first postradioiodine scintigraphy, a criterion no longer considered in the new risk stratification. The increase in the number of patients classified as “high-risk” according to the new stratification was due to the upgrade of 5.2% (n = 10) of patients initially classified as “intermediate-risk.” Among these, 7 patients were upgraded due to the presence of extranodal tumor extension, while 3 patients for tumors >4 cm associated with microscopic extrathyroidal extension. No patients classified as 2015 “high-risk” were downgraded in the ATA-risk 2025 (Fig. 2).

**Table 2:** Distribution of the 670 patients according to the 2015 and 2025 ATA risk stratifications

	Risk class	Patients, N (%)
ATA risk 2015	Low	409 (61.0)
	Intermediate	194 (29.0)
	High	67 (10.0)
ATA risk 2025	Low	318 (47.5)
	Low-Intermediate	148 (22.1)
	Intermediate-High	127 (18.9)
	High	77 (11.5)

Abbreviation: ATA, American Thyroid Association.

### Final disease outcome according to the 2 ATA risk stratifications

After a median follow-up of 53.5 months (IQR 21-120), 17.3% (N = 116) of patients had persistent disease, of whom 96 (14.3%) patients had structural incomplete response, which was managed either with a second-line treatment (surgery, RAI, or TKI therapy, n = 60), or with active surveillance (low and stable disease burden, n = 36).

**Figure 3:**
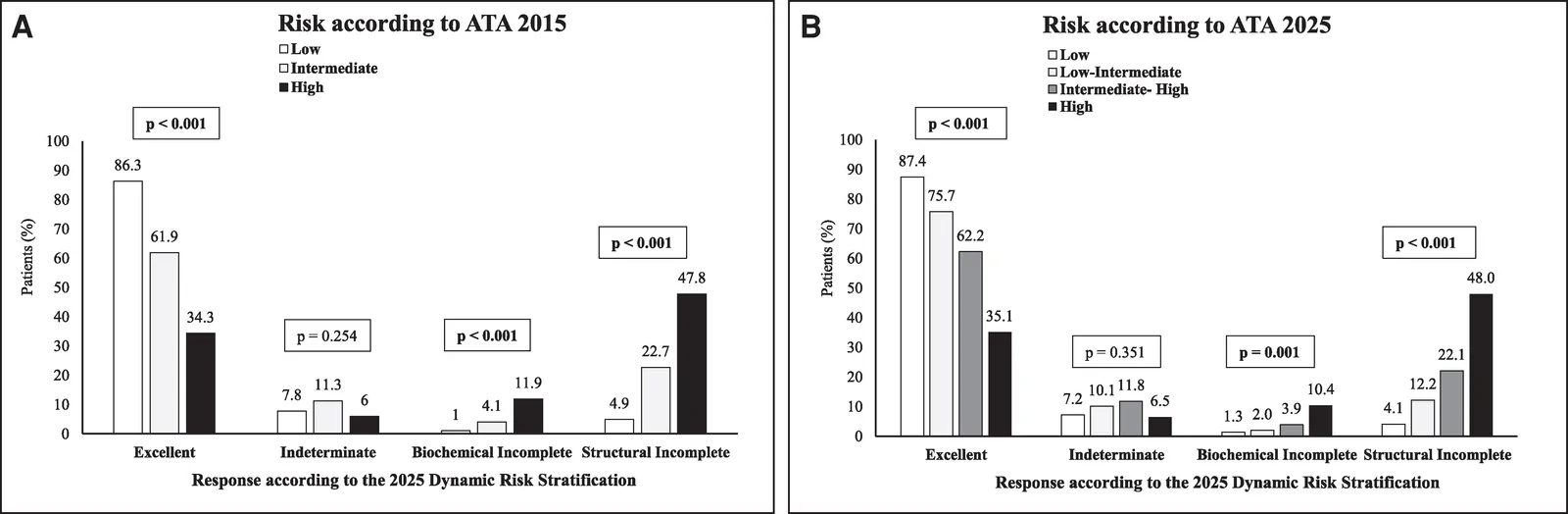
Final disease outcome for patients according to the 2015 (panel A) and 2025 (panel B) ATA risk classes.

Abbreviation: ATA, American Thyroid Association.

As shown in Fig. 3, in both the 2015 and 2025 risk stratifications, highly significant differences were found in the rate of excellent, biochemical incomplete, and structural incomplete responses according to the risk stratification, whereas no significant differences were found in the indeterminate responses.

**Figure 4:**
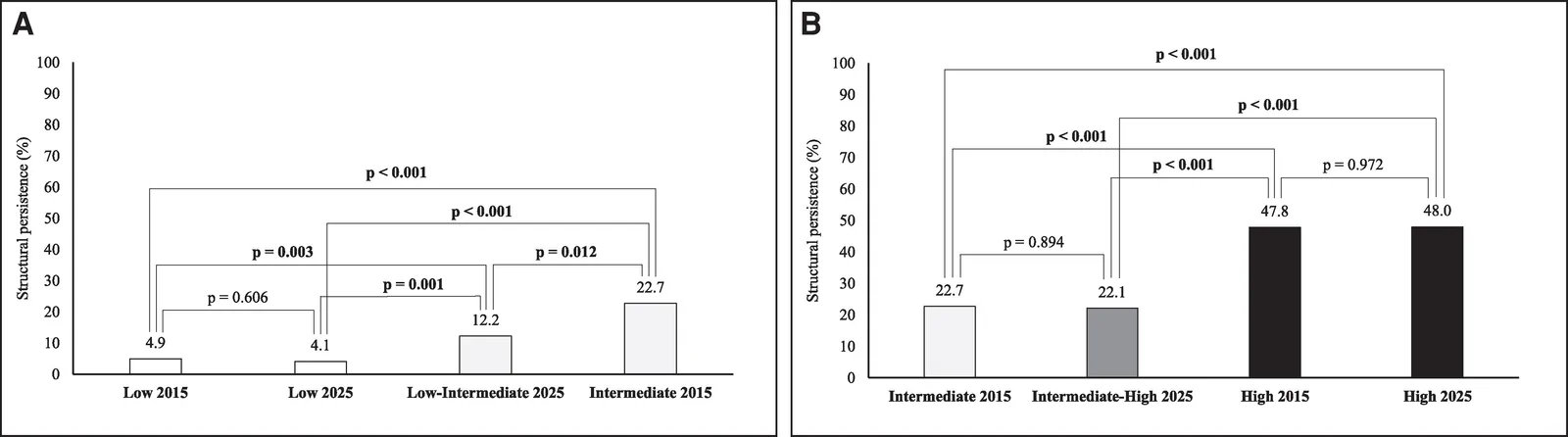
Cross-comparison of structural disease persistence between the 2015 and 2025 ATA risk classes (panel A and B).

A cross-comparison of final disease outcomes between the risk classes of the two stratifications showed that the 2025 “low-risk” class did not differ in terms of structural persistence compared to the 2015 “low-risk” class (4.1% vs 4.9%, *P* = .606). On the other hand, both the 2015 and the 2025 “low-risk” classes exhibited a lower rate of structural persistence compared to both the 2015 “intermediate-risk” class (4.9 and 4.1% vs 22.7%, *P* < .001) and the 2025 “low-intermediate-risk” class (4.9 and 4.1% vs 12.2%, *P* = .003 and .001). Interestingly, the 2025 “low-intermediate-risk” class had a lower rate of structural persistence than the 2015 “intermediate-risk” class (12.2% vs 22.7%, *P* = .012) (Fig. 4, panel A).

Neither the 2015 “intermediate-risk” class compared to the 2025 “intermediate-high-risk” class nor the 2015 and 2025 “high-risk” classes showed significant differences in structural persistence (22.7 vs 22.1%, *P* = .894 and 47.8 vs 48.0, *P* = .972). Both the “intermediate-risk” and “intermediate-high-risk” classes showed highly statistically significant differences with the 2015 and the 2025 “high-risk” classes (*P* < .001) (Fig. 4, panel B).

### Subgroup analysis of patients reclassified into a different risk class

We performed a sub-analysis to assess disease persistence in patients who were reclassified into a different risk class, according to 2015 and 2025 ATA risk stratification. Among patients classified as 2015 “low-risk,” disease persistence was more frequent in those reclassified as 2025 “low-intermediate-risk” (8/91, 8.8%), compared with those who remained “low-risk” in the ATA-risk 2025 (12/315, 3.8%), although the difference did not reach statistical significance (*P* = .093). Consistently, radioiodine therapy was more frequent in the upgraded cases (33/91, 36.2%) compared to those who remained in the “low-risk” class (62/315 patients, 19.7%, *P* < .001).

Differently, among patients classified as 2015 “intermediate-risk,” the rate of persistence (44/194, 22.7%) was comparable to that observed in patients reclassified as “low-intermediate-risk” (10/57 patients, 17.5%) or “intermediate-high-risk” (28/124 patients, 22.6%) according to the 2025 stratification (*P* = .407 and *P* = .983, respectively). No differences in radioiodine therapy were observed among these groups (“intermediate-risk” (127/194, 65.5%) vs both “low-intermediate-risk” (33/57, 57.9%, *P* = .296) and “intermediate-high-risk” (85/124, 68.5%, *P* = .569)).

For patients who shifted from 2015 “intermediate-risk” to 2025 “low-risk” and from 2015 “intermediate-risk” to 2025 “high-risk,” the numbers were too small to allow a reliable evaluation (n = 3 and n = 10, respectively).

## Discussion

This study is the first to assess in a real-world setting the risk of disease persistence or recurrence across the risk classes of the updated ATA-risk 2025 and to directly compare it with the 2015 version, highlighting both consistencies and clinically meaningful differences.

Due to the high incidence of low-risk DTCs, a treatment de-escalation for this class has been strongly supported in recent years (10-15). Interestingly, our data show that the variations introduced by the updated ATA risk stratification system led to a reclassification of patients toward higher risk classes, for which both a closer follow-up and additional treatments are suggested (1). Specifically, in our cohort, the proportion of patients classified as “low-risk” of recurrence was lower compared with the ATA-risk 2015 (−22.2%), with a corresponding increase in patients classified as “intermediate-risk” and “high-risk” (+41.7% and +14.9%, respectively). This shift reflects the introduction of additional variables now considered as risk factors for disease persistence or recurrence. According to the ATA-risk 2025, the presence of multifocality, tumor size > 4 cm, microscopically positive posterior margins (R1), and clinically evident lateral cervical lymph node metastases led to the reclassification of 94 patients from “low-risk” to “intermediate-risk” class. Moreover, tumor size > 4 cm associated with microscopic extrathyroidal extension, and the presence of extranodal extension led to the reclassification of 10 patients to the “high-risk” class.

Only a limited number of variables resulted in risk downgrading in our cohort. These included the presence of cervical metastases showing radioiodine uptake on the first post-therapy whole-body scan, a variable no longer considered in the updated stratification, which led to the downgrading of one patient, and the presence of lymph node metastases measuring 2 mm, now classified as a “low-risk” variable but previously included in the “intermediate-risk” class, resulting in the downgrading of 2 patients.

Stratification of patients according to disease outcome at the end of follow-up showed that both the 2015 and 2025 risk stratification systems were able to identify highly significant differences among risk classes with respect to excellent, biochemical, and structural responses, whereas no significant differences were observed for indeterminate responses. Notably, despite the reduction in the number of patients classified as “low-risk” according to the ATA-risk 2025, the proportion of patients achieving an excellent response did not increase (87.4% vs 86.3%).

Moreover, an interesting and original finding of this study came from the cross-comparison of the risk classes of the 2 stratification systems. In particular, the risk of structural disease persistence in the 2025 “low-risk” and “high-risk” classes was comparable to that of the ATA-risk 2015. Among the “intermediate-risk” classes, patients classified as 2025 “intermediate-high-risk” had a risk comparable to that of patients previously classified as “intermediate-risk.” Conversely, patients classified as “low-intermediate-risk” differed significantly from all classes in the previous stratification, exhibiting a higher risk than the former 2015 “low-risk” class and a lower risk than the former 2015 “intermediate-risk” class. Consistently, patients upgraded from 2015 “low-risk” to 2025 “low-intermediate-risk” had a higher rate of structural persistence than those who remained in the “low-risk” class, whereas tumors in the “low-intermediate-risk” group originating from the former 2015 “intermediate-risk” category may be considered downgraded. Thus, in this real-life context, the new “low-intermediate-risk” class can be confirmed as a new class, since it associates to a significantly different outcome with respect to the 2015 “low-risk” and “intermediate-risk” classes. In the updated ATA risk stratification system, this class shows a structural persistence or recurrence rate of 12.2%, placing it between the “low-risk” and “intermediate-high-risk” classes. Accordingly, radioiodine therapy was more frequent in the cases upgraded to 2025 “low-intermediate-risk” compared to those who remained in the 2015 “low-risk” class, likely because these patients were not fully cured after initial surgery. Nonetheless, the updated ATA guidelines do not offer specific recommendations for the postoperative management of this newly defined class and suggest evaluating case by case the potential benefit of radioiodine therapy.

Besides the high rate of patients' exclusion due to incompleteness of the pathological data, the main limitations of this study are the following. First, its retrospective design and the wide enrollment period, which implies that patients could have been submitted to different therapeutic approaches in terms of both surgical extent and radioiodine administration. In particular, patients diagnosed before 2015 may have been overtreated in accordance with previous guidelines. Nevertheless, these unnecessary treatments are not expected to substantially affect the final outcomes of these patients. Moreover, most of the patients enrolled have been diagnosed after 2015 and thus uniformly treated according to the ATA guidelines. A second limitation pertains to the follow-up, which may have been too short for some patients, and structural recurrence may not have occurred within the observed period. However, only patients with a minimum follow-up of 12 months have been included in this study, and the median follow-up of 4.5 years likely limited this potential underestimation.

In conclusion, we show for the first time in a real-life context that the introduction of additional risk variables in the ATA risk 2025 leads to a redistribution of patients across risk classes, with a shift toward higher-risk categories. With the validation of the “low-intermediate-risk” class for risk of structural recurrence, prospective clinical studies are needed to determine if changes in clinical care are indicated for this newly defined class.

## Data Availability

Some or all datasets generated during and/or analyzed during the current study are not publicly available but are available from the corresponding author on reasonable request at https://doi.org/10.5281/zenodo.19553336.
